# p38 MAPK Signaling in Osteoblast Differentiation

**DOI:** 10.3389/fcell.2016.00040

**Published:** 2016-05-06

**Authors:** Eddie Rodríguez-Carballo, Beatriz Gámez, Francesc Ventura

**Affiliations:** ^1^Department of Genetics and Evolution, University of GenevaGeneva, Switzerland; ^2^Departament de Ciències Fisiològiques II, Universitat de Barcelona and IDIBELL, L'Hospitalet de LlobregatBarcelona, Spain

**Keywords:** p38, MAP kinase, bone development, bone homeostasis, osteoblast, cell signaling, cell differentiation

## Abstract

The skeleton is a highly dynamic tissue whose structure relies on the balance between bone deposition and resorption. This equilibrium, which depends on osteoblast and osteoclast functions, is controlled by multiple factors that can be modulated post-translationally. Some of the modulators are Mitogen-activated kinases (MAPKs), whose role has been studied *in vivo* and *in vitro*. p38-MAPK modifies the transactivation ability of some key transcription factors in chondrocytes, osteoblasts and osteoclasts, which affects their differentiation and function. Several commercially available inhibitors have helped to determine p38 action on these processes. Although it is frequently mentioned in the literature, this chemical approach is not always as accurate as it should be. Conditional knockouts are a useful genetic tool that could unravel the role of p38 in shaping the skeleton. In this review, we will summarize the state of the art on p38 activity during osteoblast differentiation and function, and emphasize the triggers of this MAPK.

## Introduction

Two decades ago, the Mitogen-activated kinases (MAPKs) were revealed as key players in skeletal development and bone homeostasis that particularly affect osteoblast commitment and differentiation. Of the three classic MAPKs, scientific evidence predominantly points to p38 and ERK activities as determining and shaping the skeleton. Remarkably, there are hundreds of reports of *in vivo* and *in vitro* studies that analyse the relevant role of p38 and ERK throughout the osteoblastic commitment process, from a mesenchymal progenitor into a fully functional anabolic bone cell. This field of study has been facilitated by the availability of specific inhibitors of MAPK activity. However, these inhibitors may lead to dubious correlations between the specific causal effect of MAPK inhibition and MAPKs' role. There are several complete or cell-directed knockouts that provide a broader view of the many effects that MAPKs have on bone differentiation. The purpose of this review is to describe the precise role of p38-MAPK on osteoblast differentiation and the several upstream events that can trigger its activation, in the interests of guiding anabolic therapies for bone-related pathologies.

### Bone and its constituents

The skeleton is a very dynamic, calcified organ whose structure is maintained by bone deposition and resorption. During the last decade, new skeletal functions beyond those associated with locomotion and organ protection have been discovered, including fertility, glucose and adipose metabolism, phosphate renal clearance and maintenance of the hematopoietic niche (Karsenty and Ferron, 2012). Structurally, bone tissue is composed of different cells and an extracellular matrix (ECM). This matrix has two components: one organic and another inorganic. The latter is mainly formed by hydroxyapatite, which represents 99% of the body's calcium and 80% of the body's phosphate. The organic component is composed of collagen fibers, glycosaminoglycans, proteoglycans and glycoproteins. Collagen I is the most common protein in bone, accompanied by proteins such as bone sialoprotein (*IBSP*) and osteonectin (*SPARC*). Moreover, certain cytokines, such as TGF-β (transforming growth factor-β) and BMP (bone morphogenetic protein), remain bound to ECM fibers and can be freed during resorption processes (Dallas et al., 2002; Gregory et al., 2005).

Bone formation can be explained from two perspectives: embryological origin, and ossification. Embryologically, facial skeletal structures are derived from the ectoderm, while the axial and appendicular skeletons emerge from the paraxial mesoderm and the lateral mesoderm plate, respectively (Berendsen and Olsen, 2015). Ossification can be defined as endochondral or intramembranous. The latter mechanism occurs directly from mesenchymal condensation, in which mesenchymal stem cells (MSCs) differentiate into osteoblasts (Percival and Richtsmeier, 2013). Endochondral ossification is a very well-characterized process that takes place in long bones. A cartilage cast is invaded by mesenchymal progenitors, which leads to the appearance of a chondrocyte-enriched growth plate that allows longitudinal bone growth, and an osteoblast-driven ossification center in each epiphysis. Concurrently, cells in the periphery of the cartilage cast will differentiate into osteoblasts, which create the bone cortical structures (Long and Ornitz, 2013).

**Figure 1:**
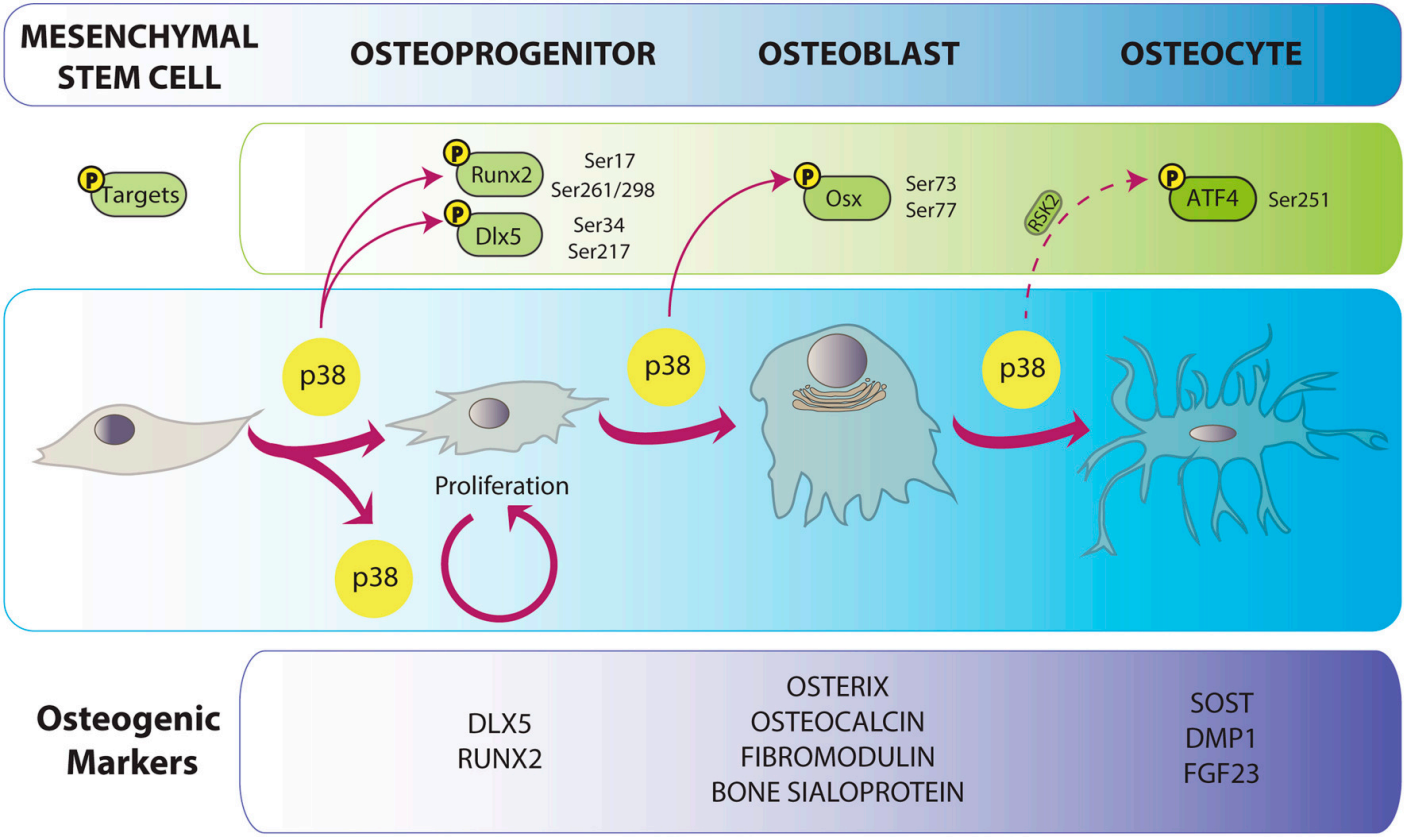
**p38 implication in osteoblast differentiation**. Osteoblast differentiation is characterized by the expression of a specific cohort of proteins depicted in the lower panel. p38 is related both to differentiation and proliferation of bone progenitors. p38-mediated phosphorylation promotes progression in osteogenesis by the enhancement of the activity or expression of osteoblast-specific transcription factors genes. Direct arrows indicate direct phosphorylation while dashed arrow indicates indirect action of p38. Phosphorylated residues are specified for every target.

Once formed, several cells from different origins end up composing the skeleton. The osteoblast is considered the most anabolic bone cell, due to its ability to secrete and calcify the extracellular matrix. Osteoblasts are cuboidal cells that lie on the surface of bone matrix (Long, 2012). They are derived from MSCs that can also differentiate into adipocytes, myoblasts, chondrocytes and fibroblasts (Augello and De Bari, 2010). Differentiation into osteoblasts is a complex process, in which serial commitment landmarks are achieved sequentially (Figure 1). It is accepted that during the first steps of commitment, these progenitors can still express stem markers and chondrocyte markers (e.g., SOX9). Pre-osteoblasts have already lost signature genes of the chondrogenic profile, and they start to express certain specific markers associated with the osteoblast linage, although they cannot produce extracellular matrix yet. These osteoblast-specific genes include transcription factors such as *RUNX2, DLX5* and *SP7* (Osterix). Soon after, other markers that are related to matrix formation start to be expressed: osteocalcin (*Bglap2*), fibromodulin (*Fmod*), and bone sialoprotein (*Ibsp*), among others (Franceschi et al., 2007; Long, 2012).

Once osteoblasts have reached the bone formation phase, four outcomes may occur: (1) they get trapped in bone matrix as osteocytes; (2) they become bone-lining cells; (3) they enter apoptosis; and (4) they can trans-differentiate into cells that depose chondroid tissue (Franz-Odendaal et al., 2006; Rochefort et al., 2010). Typically, osteocytes are closed in lacunae inside calcified bone. In the beginning of this embedding process, young osteocytes still look like secretory cells and express osteoblast markers. Some of these will be lost during terminal differentiation, while mature osteocytic markers appear (Rochefort et al., 2010). Osteocytes tend to express higher levels of *FGF23* (fibroblast growth factor 23), *NPY* (neuropeptide-Y), *RLN* (reelin), *SOST* (sclerostin), *DMP1* (dentin matrix acidic phosphoprotein 1), *PHEX* (phosphate-regulating neutral endopeptidase, X-linked), *PDPN* (gp38/E11), while matrix metalloproteinases and collagen proteins are more closely related to the osteoblast state (Paic et al., 2009). Recently, relevant functions have been attributed to osteocytes, including bone remodeling and regulation of the hematopoietic stem cell niche (Asada et al., 2013). Remarkably, osteocytes show dendritic-like cytoplasmic prolongations (50–60 per cell) that form a canalicular system inside bone (Rochefort et al., 2010; Klein-Nulend et al., 2013). These structures serve for sensing and interpreting mechanical inputs like bone loading (Rochefort et al., 2010; Xu et al., 2012).

Bone resorption is the process by which the mineral extracellular matrix is degraded. This process is primarily carried out by osteoclasts, which are derived from monocytes and macrophages (Ikeda and Takeshita, 2016). They proliferate in the bone marrow, and fuse to give rise to multinuclear reabsorbing cells close to the resorption region. Osteoclast progenitors express RANK (receptor activator of nuclear factor-κB), which interacts with RANKL (receptor activator of nuclear factor-κB ligand). Several cells produce RANKL, including osteoblasts, osteocytes, stromal cells and lymphocytes (O'Brien et al., 2013; Ikeda and Takeshita, 2016). The RANK activation process can be antagonized by osteoprotegerin (OPG), a RANKL competitor that is mainly expressed by osteoblasts (Simonet et al., 1997). Other factors contribute to the activation and function of osteoclasts, such as vitamin D and SOST, which is also mainly produced by osteocytes (van Bezooijen et al., 2005).

## The MAPK signaling pathways

### Introduction to the MAPKs

MAPKs are a family of enzymes that are implicated in a series of processes in which extracellular stimuli (e.g., environmental stress, growth factors and cytokines) are transduced into different cellular actions. In some cases, they act as a signaling hub in which different signaling pathways converge to activate a MAPK in a given time frame or tissue. Conventional MAPK members are the extracellular signal-regulated kinases 1/2 (ERK1/2) and ERK5, c-Jun amino (N)-terminal kinases 1/2/3 (JNK1/2/3), and the p38 isoforms (p38α, p38β, p38γ, and p38δ) (reviewed in Cargnello and Roux, 2011). All of them contain a Ser/Thr kinase domain, activated by phosphorylation by other Ser/Thr kinases. Thus, MAPK signaling constitutes a series of phosphorylations, in which several elements are at play until the final substrate is targeted. Therefore, once stimuli have reached the cell, MAPKK kinases (MAP3K) are activated and phosphorylate MAPK kinases (MAP2K), which in turn phosphorylate and activate the aforementioned MAPKs. Each MAPK group has its own series of upstream activators, thus each represents a specific signaling cascade (Cuadrado and Nebreda, 2010).

### JNK signaling in osteoblastic differentiation

The role of JNK in osteoblastogenesis seems to be somewhat contradictory, with different functional outputs depending on the study. For instance, interleukin-1β (IL1β) and tumor necrosis factor-α (TNFα) favor JNK activation and this effect is determinant for osteoblast differentiation of human periosteal cells (Hah et al., 2013). Moreover, Liu et al. found that inhibition of the JNK route by chemical inhibitors or siRNAs led to decreased mineralization and downregulation of several osteogenic markers, while its activation by a constitutive active form favored osteogenesis. These effects were, at least in part, due to reduced SMAD6 binding to BMPRI (BMP receptor I), which allows SMAD1 to access the receptor (Liu et al., 2011). However, another effect of JNK activation might be to negatively regulate osteogenesis. JNK1 reduces RUNX2 transcriptional activity by phosphorylation at Ser104 (Huang et al., 2012). In addition, the inhibition of JNK with SP600125 in human MSC increases ALP activity induced by BMP-2 (Biver et al., 2014). This inhibitory action may also integrate SMAD1 signaling, as MAPK phosphorylation is necessary for GSK3β (Glycogen synthase kinase 3β)-induced SMAD1 degradation (Fuentealba et al., 2007; Biver et al., 2014).

### ERK signaling in osteoblastic differentiation

Both ERK1 and ERK2 are expressed in osteoblasts and have relevant functions in bone metabolism. There is genetic evidence of the implication of the ERK pathway in osteogenesis. First, the expression of a dominant negative form of MEK1 under the regulation of the osteocalcin promoter (mOG2:*Mek1*DN) exhibits calvarial and clavicular defects, which phenocopy the *Runx2* deficiency. The phenotype of *Runx2*^+/−^ is recovered by crossing these mutant mice with a constitutive active form of MEK1 (mOG2:tg*Mek1*SP) (Ge et al., 2007). Matsushita and collaborators generated the double mutant *Erk1*^−/−^*;Erk2*^Prx1:Cre^. They showed the positive effect of ERK1 and ERK2 on osteoblast differentiation, and the inhibitory effect of these MAPKs on chondrocyte differentiation at the perichondrium. In addition, ERK has certain effects on RANKL in these cells, which has an impact on osteoclast activation (Matsushita et al., 2009).

RUNX2 phosphorylation is the action of ERK signaling on osteoblast specification that has been most widely studied. First, use of the MEK1 inhibitor PD98059 blocks osteocalcin induction by RUNX2 (Xiao et al., 2000). U0126, another MEK1/2 inhibitor, halts the expression of osteocalcin and *Ibsp* mediated by BMP7 and ascorbic acid (Xiao et al., 2002a). In addition, FGFs activates pERK, and subsequently a phosphorylated form of RUNX2 is detected. Again, these effects can be blocked by U0126 (Xiao et al., 2002b). Finally, the same group proved the existence of four serine residues (Ser43, Ser301, Ser319, and Ser510) targeted by ERK1/2. Ser301 and Ser319 were found to be responsible for RUNX2 activating abilities (Ge et al., 2009). The increase in pRUNX2 transcriptional activity induced by ERK could be attributed to the binding of cofactors such as CREB, CBP/p300 or vitamin D receptor (Sierra et al., 2003), as seen in p38 phosphorylation (Greenblatt et al., 2010). Related to this, an increase in cytoplasmatic pERK has been associated with an increment in nuclear RUNX2 upon overexpression of the transmembrane glycoprotein CD99, which implies that RUNX2 phosphorylation takes place in the cytoplasm (Sciandra et al., 2014).

NF1 (neurofibromatosis type I) is a GTPase-Activating Protein that turns off Ras action on ERK signaling. The inactivating mutation of *NF1* seems to have different results depending on the developmental status at which deletion takes place (Greenblatt et al., 2013). In fact, analysis of the osteoblast-specific mutant of *Nf1* shows a blunted response to BMP-2 that is overcome when ERK was inhibited by U0126 (de la Croix Ndong et al., 2015). Besides differentiation, ERK is related to the activity of cyclinD1, enhanced by PTH and PTHrP, and favoring osteoblast proliferation (Datta et al., 2007). Recently, it has been shown that ERK regulates the antiapoptotic and proliferative effects of EGF on osteoprogenitors (Chandra et al., 2013).

### p38 MAPK cascade

**Figure 2:**
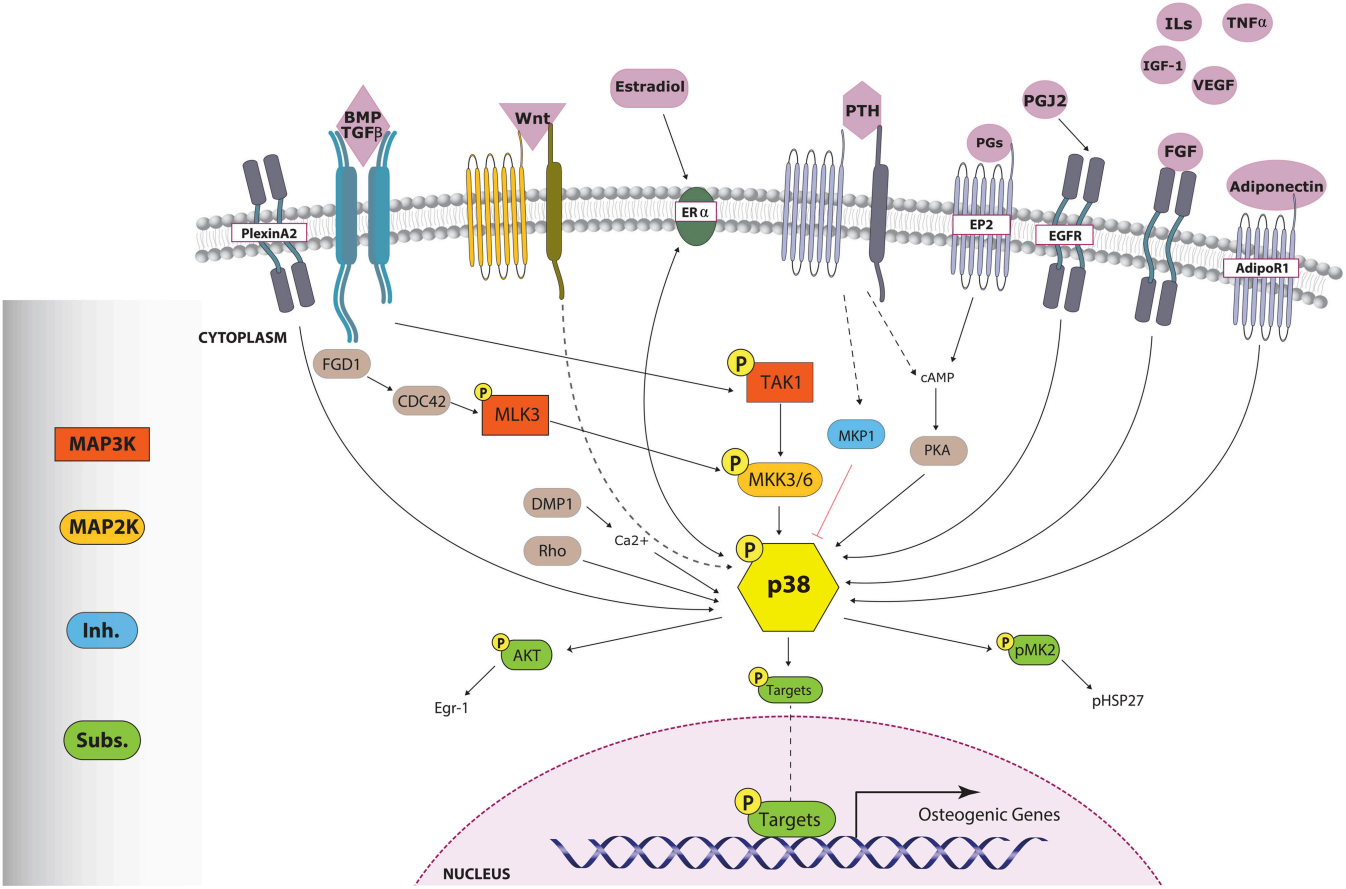
**p38 triggers involved in osteogenesis**. Numerous stimuli, through distinct mechanisms, lead to p38 MAPK phosphorylation. Activated p38 phosphorylates important osteoblast-related transcription factors, which will enhance osteogenic gene expression. pp38 can also trigger other pathways through the activation of cytoplasmic-acting substrates such as AKT and pMK2. Sharp arrows indicate direct activation. Dashed arrows indicate an unknown or indirect mechanism of action while the red flat-tipped arrow denotes inhibition.

The p38 MAPK family is composed of four proteins: p38α (encoded by the gene *Mapk14*), p38β (*Mapk11*), p38γ (*Mapk12*), and p38δ (*Mapk13*). Their coding genes have a distinct tissue distribution and they appear differentially expressed, being *Mapk14* the most highly expressed (Cuadrado and Nebreda, 2010). p38 MAPKs are substrates for three MAP2K (MKK6, MKK3, and MKK4). The contribution of each of these MAP2K to p38 MAPKs activation depends on the stimulus and the cell type (Alonso et al., 2000; Brancho et al., 2003; Remy et al., 2010; Figure 2). The MAP3Ks that lead to p38 MAPKs activation are ASK1, DLK1, TAK1, TAO1, TAO2, TPL2, MLK3, MEKK3, MEKK4, and ZAK1 (Cuadrado and Nebreda, 2010). p38α can also be autophosphorylated, due to activation through ZAP70, p56^*lck*^, and TAB1, and downregulation of Cdc47 (Cuadrado and Nebreda, 2010).

It is known that there are several scaffold complexes along this route that facilitate MAPK-MAP2K interaction or locally increase the concentration of effectors of the MAPK cascade, driving fine spatio-temporal regulation. The most commonly reported molecules related to this function belong to the protein family named JIP (from c-Jun NH2-terminal kinase-interacting protein) that can tether p38 and JNK to their upstream MAP2K. JLP, which have the isoforms JIP4 and SPAG9 (Kelkar et al., 2005; Dhanasekaran et al., 2007) and JIP2, have been implicated in regulating p38 by tethering to MKK3, MEKK3, and MKK4 (Dhanasekaran et al., 2007). The role of these scaffold proteins in regulating MAP2K action could be of special interest, as it has been shown that constitutive activation of MKK3 or MEK1 has deleterious effects on BMP-induced osteoblastogenesis (Huang et al., 2014). Apart from JIP proteins, there are other potential MAPK scaffolds: RACK1 (Arimoto et al., 2008), KSR-2 (Liu et al., 2009), Sec8 (Tanaka et al., 2014) and the osmosensing scaffold for Rac-MEKK3-MKK3 (OSM) (Dhanasekaran et al., 2007). Out of these, *Mapk8ip2* (JIP2) is detected in the developing skeleton, and *Exoc4* (Sec8 protein), which interacts with JIP4, is expressed in bone cells (www.genecards.org and www.eurexpress.org). Besides the aforementioned proteins, TAK1 and TAB1 could also be considered a sort of scaffold proteins, as they facilitate the recruitment and activation of upstream effectors of the p38 pathway. However, no MAPK scaffold proteins have been investigated in osteoblast development *in vivo* to date.

p38 MAPKs are inactivated mainly by dephosphorylation by certain phosphatases belonging to the DUSP family (Bermudez et al., 2010). Moreover, the catalytic activity of p38α is modulated according to how many and which threonine or tyrosine residues are phosphorylated in the activation loop (Zhang et al., 2008). Like any other relevant molecule, p38 is also regulated by mechanisms such as acetylation, protein degradation and stabilizing cofactors and, obviously, changes in the expression of their coding genes (reviewed in Cuadrado and Nebreda, 2010). p38α is localized in the nucleus and the cytoplasm (Raingeaud et al., 1995). Its localization depends on activation and on active transport, as p38 does not have a nuclear localization signal. It is known that DNA damage favors phosphorylation and nuclear accumulation of p38α, probably by releasing p38α from TAB1 or MK2, as they act as cytoplasmatic anchors (Wood et al., 2009).

## p38 and mesenchymal differentiation

The p38 pathway has been implicated in the differentiation of several mesenchymal cells. Most reports on this topic have been cell-based studies in which activation or inhibition of the pathway was achieved mainly pharmacologically.

### Adipocyte differentiation

There is some controversy about the role of p38 in adipogenesis. Some authors claim that it depends on the species or on the specific cell type used *in vitro*, where different factors could be at play. The generation of a tissue-specific knockout would shed light on this theme. p38 was linked to the phosphorylation of C/EBPβ and the expression of PPARγ (peroxisome proliferator-activated receptor-γ) in 3T3-L1 cells (Engelman et al., 1998). In fact, the overexpression of an active form of MKK6 favors adipocyte differentiation (Engelman et al., 1999). (2S)-7,4′-dihydroxy-8-prenylflavan also stimulates the adipogenic program and glucose uptake via p38 (Ji et al., 2015). Contrary to the main current of knowledge regarding BMP-directed osteogenesis, this cytokine also stimulates adipogenesis of mesenchymal precursors via p38, probably involving SMAD proteins as well (Hata et al., 2003; Huang et al., 2009). Regarding adipocyte physiology, one of the few experiments performed on living animals was the administration of the p38 inhibitor FR167653. This treatment significantly reduced weight gain, fat depots and adipocyte size (Maekawa et al., 2010). p38 also seems relevant for brown adipocyte commitment, as seen with DUSP10 induction (Choi et al., 2013) or the inhibitory effects of silica nanoparticles (Son et al., 2015). Indeed, NPY seems to affect the brown fat gene program and reduce p38 and CREB phosphorylation (Wan et al., 2015).

Other studies describe contrary effects. For example, p38 activates CHOP, a negative regulator of C/EBPβ (Wang and Ron, 1996), and also blocks NFATc4, which is a transcription factor for *Pparg* (Yang et al., 2002). In chicken adipocytes, adiponectin prompts a cascade through p38/ATF2 that inhibited CEBPα, and affects preadipocyte differentiation (Yan et al., 2013). Adipogenesis conducted by microtubule affinity-regulating kinase 4 (Marc4) depends on JNK activation and p38 downregulation in 3T3-L1 (Feng et al., 2014). p38 activity seems to decrease during adipocyte differentiation, and inhibition of p38 augments PPARγ transactivation and other adipocyte markers. Furthermore, p38-deficient MEFs have increased expression of adipogenic markers such as adiponectin or leptin (Aouadi et al., 2006). Strikingly, the same group proposed a positive role for p38 during human adipocyte differentiation, mainly based on C/EBPβ phosphorylation (Aouadi et al., 2007). Ferguson et al. linked the role of DUSP1 phosphatase to the deactivation of p38 and ERK during the first steps of preadipocyte commitment, which establishes a framework for self-regulatory feedback (Ferguson et al., 2016).

### Myoblast differentiation

p38 MAPK isoforms have multiple roles in muscle development and homeostasis in the adult. They control self-renewal, proliferation, asymmetric division and differentiation of satellite cells (Brien et al., 2013; Bernet et al., 2014). Early studies show higher p38α, β, and γ levels during myoblast maturation, and that p38 inhibition blocks muscle-specific gene transcription (Cuenda and Cohen, 1999; Zetser et al., 1999). p38 activity is absolutely required for the transition from proliferating satellite cells into terminally differentiated myoblasts (Puri et al., 2000). This control of the muscle-specific gene program is performed through multiple steps that finally lead to activation of key myogenic transcription factors. For instance, p38 induces the transcriptional activity of MEF2 family members through direct phosphorylation at Thr293 (Wu et al., 2000; Lluis et al., 2006). In addition, p38 also phosphorylates and activates the obligate MyoD partner E47 (Lluis et al., 2005). Several reports indicate that p38 activity is required for chromatin remodeling, to allow access and stabilization of the binding of myogenic transcription factors to myogenic loci (Lluis et al., 2006). These mechanisms involve histone acetylation by PCAF and p300, and recruitment of the SWI/SNF complex (Simone et al., 2004; de la Serna et al., 2005). Subsequently, p38 activity is important for myocyte fusion and myofibrillogenesis (Gardner et al., 2015).

More recently, the role of p38 in muscle development has been extended to the activation of satellite cells during muscle regeneration. Muscle regeneration takes place in a highly inflammatory environment, and inflammatory cytokines such as IL6 or TNFα activate p38 (Li et al., 2014c). Signaling from p38α/β is involved in the exit of satellite cells from quiescence (Jones et al., 2005; Brien et al., 2013), and arranges asymmetric division and self-renewal (Troy et al., 2012; Bernet et al., 2014). Importantly, skeletal muscle aging results in a loss of muscle mass and regenerative capacity. These defects arise because satellite cells from aged mice fail to self-renew and increase p38 activity. Since pharmacological manipulation of p38α/β activity ameliorates these age-associated defects, this could be a potential therapeutic opportunity to treat muscle wasting (Bernet et al., 2014).

### Osteoblast differentiation

One of the first studies to indicate that p38, ERK, and JNK were activated during osteoblast differentiation of human MSC was Jaiswal et al. (2000). Gallea and collaborators showed that ERK and p38 activation in C2C12 favored osteoblast determination (Gallea et al., 2001). Soon after, it was shown that the inhibitor SB203580 impairs MC3T3 pre-osteoblast differentiation and the expression of osteoblast markers such as ALP, OC, and collagen (Suzuki et al., 1999, 2002). As will be seen later, many stimuli have been assayed as triggers of p38 activation in osteoblasts, to explore their osteogenic activities.

## The study of p38 in bone: different strategies, the same goal

### Knockouts of p38

Different groups have generated germline knockout mice in which the p38 pathway is mutated. It should be stated that p38α is the most highly expressed isoform of p38-MAPK in osteoblasts (Greenblatt et al., 2010; Rodriguez-Carballo et al., 2014). Embryos with a homozygous deletion of *p38a*, as well as embryos with a double knockout of *Mkk3/Mkk6*, die during embryogenesis, while mutations of *Map2k3* (MKK3), *Map2k6* (MKK6), *Mapk11, Mapk12*, and *Mapk13* are viable (Cuadrado and Nebreda, 2010). Both *Map2k3*^−/−^ and *Map2k6*^+/−^ display severe skeletal defects in long bones, while only *Map2k3*^−/−^ show abnormalities in craniofacial bone structures (Greenblatt et al., 2010). The embryonic lethality of *Mapk14* loss is associated with neural and cardiac defects (del Barco Barrantes et al., 2011). Initially, a lack of *Mapk11* was not implicated in severe defects (Beardmore et al., 2005), although later it has been associated with some mild bone defects (Greenblatt et al., 2010). The *Mapk13*^−/−^ mice has reduced sensitivity to skin carcinogenesis (Schindler et al., 2009), as well as higher insulin secretion and tolerance to glucose (Sumara et al., 2009). No bone phenotype was described in the original article in which *Mapk12*^−/−^ and *Mapk13*^−/−^ were generated (Sabio et al., 2005), or in successive studies.

In the last 5 years, three studies reported the use of bone conditional knockouts affecting the p38 pathway in osteoblasts (reviewed in Thouverey and Caverzasio, 2015a). Greenblatt and collaborators' integral approach involved analysing the deletions of different effectors of the pathway. The conditional deletion of MAP3K TAK1 in pre-osteoblasts leads to defects in osteoblast differentiation and bone formation that affects the entire skeleton. In fact, the phenotype resembles human cleidocranial dysplasia, which is related to RUNX2 mutations. Indeed, one of the conclusions of the study is that RUNX2 is a target of p38 kinase activity, which is essential to the transactivation function of RUNX2 (Greenblatt et al., 2010). Craniofacial defects are mainly associated with the function of TAK1, MKK3, and p38α, particularly when it affects pre-osteoblasts, as with the use of an Osterix-driven recombinase (Greenblatt et al., 2010; Rodriguez-Carballo et al., 2014). No cranial phenotype was described when an osteocalcin-Cre animal model was used (Thouverey and Caverzasio, 2012). Additionally, with the latter approach, a post-natal bone acquisition defect starts only after 5 weeks of age and disturbs trabecular and cortical bone volumes of mice (Thouverey and Caverzasio, 2012). A doxycycline-induced recombinase can be used to monitor the impact of p38α deletion in osteochondroprogenitors at different time points. When the deletion starts at 3 weeks of age, the anabolic defects are mainly trabecular at 30 weeks of age, and cortical at 60 weeks. When the deletion starts in young adults (circa 8 weeks of age), only cortical defects are encountered. This means that p38α function is particularly important at different stages of osteoblast commitment throughout life (Rodriguez-Carballo et al., 2014). Interestingly, this low bone mass model was also used to hypothesize about the crosstalk between the skeleton and the adipose tissue (Rodriguez-Carballo et al., 2015).

### Pharmacological inhibitors of p38

The use of knockout models represents the most faithful, general analysis of MAPK activity on tissues. Primary osteoblast cultures from these knockout animals, or the manipulation of pathway effectors (via constitutive activation or dominant negative forms), allows molecular analysis in specific models. However, the most frequent approach is the use of selective inhibitors of the different MAPKs. The most common p38 inhibitor is the pyridinyl imidazole molecule SB203580 (Lee et al., 1994; Cuenda et al., 1995). This is considered a highly selective p38α/β inhibitor and has been widely used for more than 20 years (it had over 6000 PubMed entries up to 2015). Nevertheless, it has been shown that it can inhibit other kinases, such as GAK, CK1, RIP2, RAF, and GSK3, and the formation of ZMP from AICAR (Bain et al., 2007). The SB203580 inhibitor has been assayed in some inflammatory skeletal conditions, such as arthritis (Badger et al., 1996). BIRB0796 is even more powerful at blocking p38α/β, but at moderate doses it can also halt the activity of p38γ, p38δ, and JNK2α2 (Bain et al., 2007). SD-822 is considered a more selective p38α inhibitor (Koppelman et al., 2008) and has been used in studies of osteoarthritis to reduce the outcome of the disease (Medicherla et al., 2006). Caverzasio and colleagues showed the potential benefits of this inhibitor for the treatment of osteoporosis, as it reduces osteoclast activity (Caverzasio et al., 2008).

### p38 osteogenic targets

The osteogenic potential of p38 kinase is related to its capacity to phosphorylate and increase the activity of some key osteogenic transcription factors. As explained above, both ERK and p38 can phosphorylate RUNX2, boosting its transcriptional potential (Ge et al., 2009, 2012; Greenblatt et al., 2010; Artigas et al., 2014). In addition, our group has described new p38 phosphorylation targets in recent years. For instance, BMP-2 stimulus increases DLX5 transactivation of the *Sp7* (*Osx*) promoter through a feed-forward mechanism. First, *Dlx5* mRNA levels are elevated by BMP treatment (Miyama et al., 1999; Luo et al., 2001; Holleville et al., 2003; Ulsamer et al., 2008; Rodriguez-Carballo et al., 2011). Then, post-translationally, DLX5 is phosphorylated by p38 MAPK at serines Ser34 and Ser217, which facilitates the recruitment of p300 (Ulsamer et al., 2008; Figure 1). Another DLX member, *Dlx3*, is also induced by BMP-2. In this case, it occurs through cooperation between SMAD5 and pp38 as they translocate to the nucleus and exert their function on the *Dlx3* promoter (Yang et al., 2014). These p38-activated osteogenic events continue with the phosphorylation of Osterix at serines Ser77 and Ser33. As for DLX5, these modifications represent an increase in the transcriptional ability of OSX by helping to recruit p300 and BRG-1 (Ortuno et al., 2010, 2013). It was also shown that OSX can cooperate with RUNX2 to induce *Col1a1* (Ortuno et al., 2013). In fact, soon after, it was proven that indeed OSX and RUNX2 bind each other and cooperate to increase their transcriptional power. This interaction requires the action of p38 and ERK MAPKs, as mutation of the phosphorylation sites of RUNX2 and OSX prevented their interaction (Artigas et al., 2014).

In addition to these classic osteogenic targets, p38 can phosphorylate ATF members at threonines Thr69 and Thr71, as well as CREB at the serine Ser133. Both ATF and CREB are transcriptional co-factors and, again, these phosphorylations increase their transactivation capacity, including the recruitment of histone acetyltransferase p300 (Livingstone et al., 1995; Waas et al., 2001). More importantly, ATF4 phosphorylation by RSK2 has been shown to be absolutely required for bone development (Yang et al., 2004). Both ERK and p38 turn on RSK2 in different cell types (Siebel et al., 2013; Czaplinska et al., 2014), which then can lead to activation of ATF4 by its phosphorylation at Ser251 (Yang et al., 2004).

#### Signaling interaction between MAPKs in osteoblasts

Very few studies have focused on the degree of interaction between different MAPK pathways. The crosstalk between pathways can be both opposed and cooperative. As a rule of thumb, inhibition of one MAPK pathway activates the other. The dominant negative form of Mek (MekDN) favors the activation of p38, while the constitutively active form has the opposite effects. The same goes for ERK when Mkk6dn and Mkk6sp are used (Ge et al., 2012). Nevertheless, blocking of both pathways by SB203580 and U0126 has deleterious consequences on calvaria mineralization in organotypic cultures (Ge et al., 2012). Similarly, some reports showed that osteogenic effects require cooperation between JNK and p38 signaling (Guicheux et al., 2003).

### p38 and cell migration

Other actions besides participation in cell proliferation and differentiation are attributed to p38 MAPK, including control of cell migration. This function is vital to understand p38 MAPK's role in physiological processes, such as chemotaxis, fracture healing and wound closure, as well as the consideration of p38 as a target to diminish the invasiveness of cancer cells. The first model in which p38 was associated with cell motility was endothelial cells. SB203580 inhibited VEGF-induced cell migration (Rousseau et al., 1997). Subsequently, p38 was shown to be essential to cell migration in a plethora of different cell types. In other studies, p38 is negatively linked to cell motility. The loss of p38δ has been associated with an increase in cell motility and proliferation of squamous cancer cells (O'Callaghan et al., 2013). In C2C12, interleukin-17 inhibits cell migration by downregulating urokinase through p38α activation (Kocic et al., 2013).

As said, the positive link between p38 and cell movement appears evident. In skeletal cells, p38 activation is positively related to cytoskeletal reorganization and stimulation of cell motility. In 2004, it was reported that PDGF stimulated the proliferation and migration of MC3T3 through different MAPKs. This migration halted when p38 was inhibited, but not when JNK or ERK were blocked (Mehrotra et al., 2004). The flavonoid quercetin impairs cell migration in osteoblastic cells, but the inhibition of ERK and p38 stops this effect (Nam et al., 2008). Our group studied the function of BMPs as chemotactic agents and as inductors of actin cytoskeletal reorganization in mesenchymal cells. These events require the activation of two pathways: PI3K/CDC42/LIMK1 and p38/MAPKAP2/HSP25. Moreover, in fibroblasts depleted for *Mapk14* or *Mapkapk2*, BMP-2 signaling could not favor actin cytoskeletal reorganization and induction of movement (Gamell et al., 2008, 2011). Another interesting aspect of p38 in osteoblastic matrix remodeling is attributed to its role in a model of collagen contraction (Parreno and Hart, 2009).

Fracture assays can be performed *in vivo* and *in vitro*. In a model of fracture healing in rat, TNFα stimulates the proliferation and migration of bone marrow MSCs to the fracture site. This effect was explained by the activation of p38 and inhibition of differentiation in this area, as shown *in vitro* (Zhou et al., 2006). These migratory effects of TNFα were corroborated *in vitro* with MSCs (Fu et al., 2009).

PTH treatment stimulates amphiregulin in osteoblasts. This osteoblast expression enhances the migration and recruitment of close-by mesenchymal progenitors, due to signaling on EGFR through PI3K and p38 (Zhu et al., 2012). SDF-1 (stromal cell-derived factor-1) is described as an inductor of migration of umbilical cord MSCs through activation of AKT, ERK and p38 (Ryu et al., 2010). In fact, shear stress stimulation of JNK and p38 signaling can also provoke SDF-1 secretion in human mesenchymal cells, which activates the CXCR4 receptor and favors migration (Yuan et al., 2012).

## Signaling triggers p38 in osteoblast function

The great variety of stimuli that can activate MAPK and the extended use of specific inhibitors have facilitated the evaluation of the MAPK role in osteogenesis (Figure 2). These factors make it easier to design experimental studies to validate different osteogenic treatments. However, conclusions about the specific action of a given treatment that is supposed to affect MAPK signaling should be treated with caution. First, inhibition of a MAPK could favor the activation of another MAPK. Secondly, different inhibitors have associated effects that have not been characterized fully. Third, MAPK pathways are stress-activated cascades, and if there are no proper controls, osmotic stress could account for part of the related effects. And fourth, too often researchers do not ascertain which mechanism activates p38.

### Osteogenic hormones and growth factors

#### p38 is a non-canonical TGFβ/BMP signaling pathway

The stimulation of p38 by BMP through TAK1 was first observed in the PC12 cell line (Iwasaki et al., 1999). Then, it was shown how BMP and TGFβ activate p38, ERK and JNK in different mesenchymal cells, including osteoblasts. The involvement of these MAPKs has been observed in different osteogenic *in vitro* models (Gallea et al., 2001; Viñals et al., 2002; Guicheux et al., 2003). A recent report showed that p38 was predicted to account for 20% of the phospho-residues identified after treatment of MSCs with BMP (Halcsik et al., 2013). TGF-β provokes the phosphorylation of p38, ERK and JNK in a very rapid manner in MC3T3 and primary osteoblasts (Arnott et al., 2008). p38 and ERK are needed for *Col3a1* expression in UMR cells under BMP or TGF-β treatment (Selvamurugan et al., 2004). In addition, p38 is necessary for TGF-β-induced synthesis of VEGF in MC3T3 (Tokuda et al., 2003). Although essentially independent, there is a certain level of cross-regulation between SMADs and p38. For example, in human osteoblast cells, inhibition of p38 by SB203580 seems to make SMAD1 phosphorylation and its nuclear accumulation difficult (Noth et al., 2003). In addition, both signaling pathways are needed for BMP5 induction of limb development (Zuzarte-Luís et al., 2004). Nevertheless, p38 and ERK should be inhibited by BMP4 to guarantee self-renewal and stemness (Qi et al., 2004).

**Figure 3:**
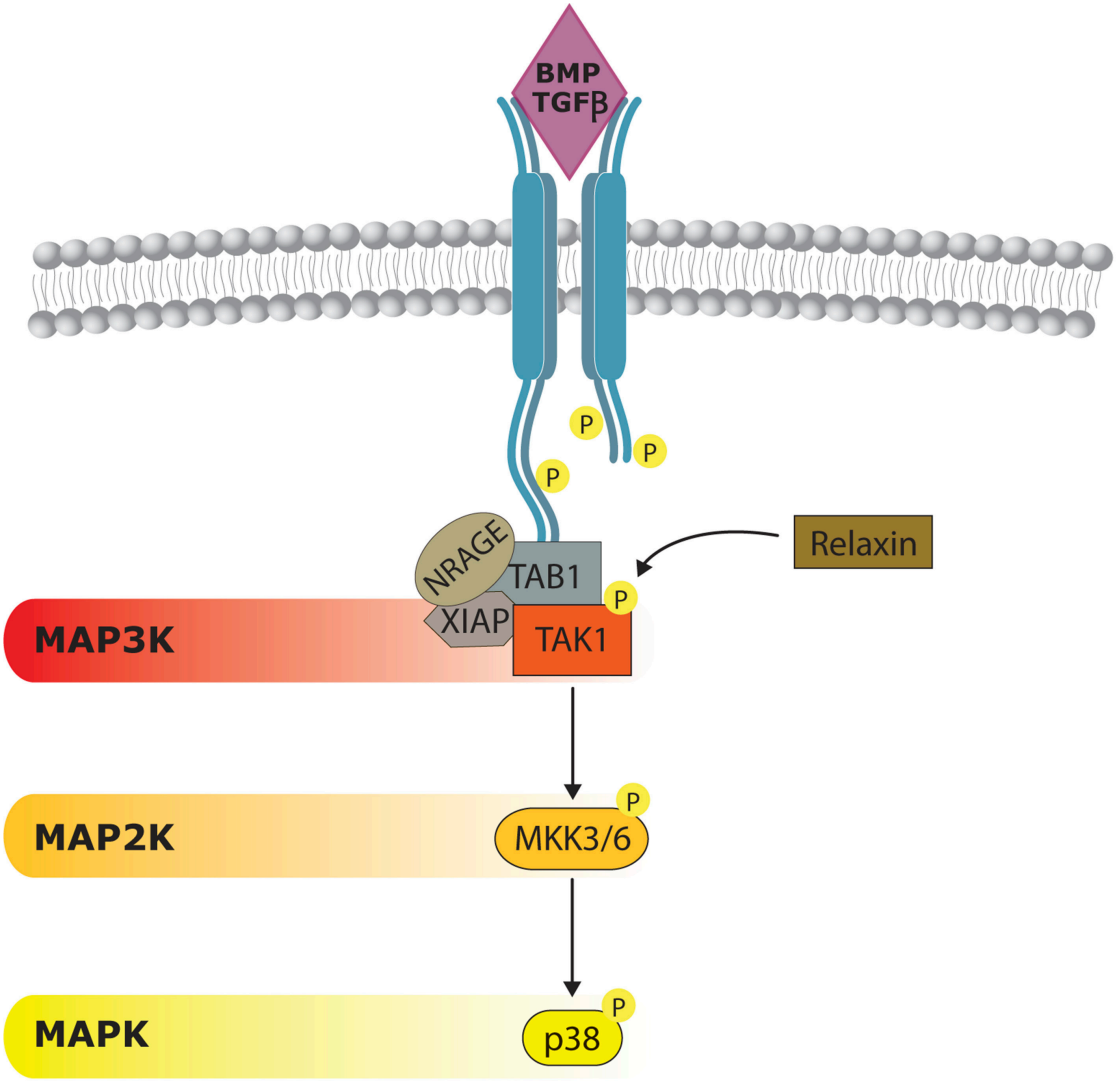
**Non-canonical BMP/TGFβ signaling**. p38 activity can be triggered by a BMP-dependent mechanism. Once BMP receptors (BMPR) are stimulated they form a complex formed by NRAGE, XIAP, and TAB1, which is responsible of TAK1 activation. Relaxin can act on TAK1, cooperating on p38 activation. Although considered to be independent from SMAD activity, some cross-regulation have also been reported.

BMP signal transduction through p38 has been studied in several cell models. As mentioned previously, the onset of the cascade begins, as for SMADs, at BMP receptor (BMPR) level. Both SMADs and p38 need the kinase activities of BMP receptor complexes. After receptor triggering, MAP3K TAK1 is activated. Mechanistically, it is well-established that TGF-b regulates the TAK1/p38 pathway through recruitment and ubiquitylation of TRAF6 by activated receptor complexes (Sorrentino et al., 2008; Yamashita et al., 2008; Figure 3). BMPRs form a complex with NRAGE, TAK1, XIAP, and TAB1, which favor p38 activation (Yamaguchi et al., 1999; Kendall et al., 2005). Interestingly, a member of the insulin/Rln family, named Relaxin, can phosphorylate TAK-1 and cooperate with BMP-2 in promoting osteogenesis by enhancing the BMP activation of the p38 signaling pathway (Moon et al., 2014).

#### Wnt signaling

**Figure 4:**
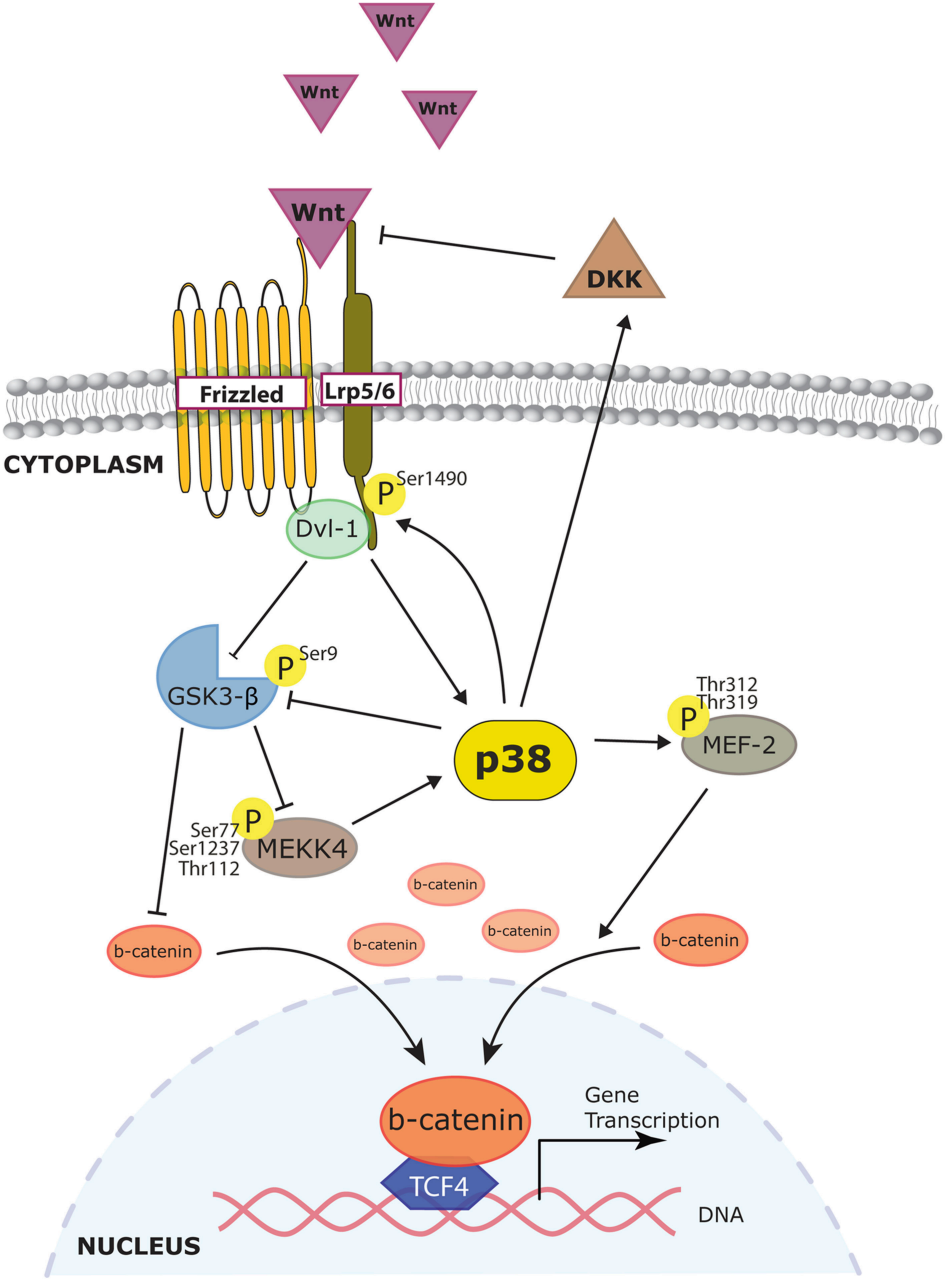
**Role of p38 MAPK in Wnt signaling**. Wnt pathway activation leads to β-catenin accumulation, which enables β-catenin to translocate and initiate the transcription of target genes. p38 participates in the regulation of β-catenin accumulation by targeting different steps of the Wnt signaling. Wnt can activate p38 through Disheveled (Dvl) proteins and at the same time also inactivate GSK3β allowing β-catenin to accumulate. Activated p38 can reinforce GSK3β inhibition therefore increasing cytoplasmic β-catenin. Moreover, effects of p38 phosphorylation on MEF2 also enhance β-catenin nuclear accumulation. p38 also exerts negative effects on Wnt signaling since DKK-1 expression (a Wnt inhibitor) relies on p38 activation.

The Wnt pathway interacts with p38 at different levels. Wnt can activate p38 through Disheveled proteins, as the silencing of *Dvl-3* avoids Wnt3a-induced ATF2 phosphorylation (Bikkavilli et al., 2008). At the same time, p38 can reinforce the Wnt/β-catenin pathway. In COS-7 and endodermic F9 cells, p38α inactivates GSK3β by phosphorylating its Ser9, allowing β-catenin to accumulate. Indeed, blocking the p38 pathway affects the triggering of Wnt3a downstream events (Bikkavilli et al., 2008). The activation of both p38 and ERK by Wnt3a, and its effects on pre-osteoblasts' commitment without disturbing proliferation, have also been reported (Caverzasio and Manen, 2007). In dental pulp cells, BMP-2 facilitates *Lef1* expression and β-catenin nuclear accumulation. The authors claimed that this was due in part to p38, as the inhibitor SB20350 prevented these effects (Yang et al., 2015b). Very recently, Ehyai reported that p38-mediated phosphorylation on MEF2 enhances β-catenin nuclear accumulation (Ehyai et al., 2015). The convergence of both pathways has also been demonstrated at the level of the WNT receptor LRP6. In HEK 293, LRP6 phosphorylation by the MAPKs p38, ERK1/2, and JNK is key for the recruitment of the multiprotein degradation complex that includes GSK3β (Cervenka et al., 2011; Figure 4). This reciprocal interaction has a negative feedback loop. GSK3β inhibits the activation of p38 and JNK by binding the MAP3K MEKK4. It was proposed that GSK3β could potentially phosphorylate an N-terminal residue of MEKK4, but this has not been proven yet (Abell et al., 2007). Another indirect negative loop has been proposed in several models: the expression of Wnt inhibitor DKK-1 relies on p38 activation, as shown in primary osteoblasts (Kamiya et al., 2010; Figure 4).

#### Estrogen receptors

Estrogen receptor (ER) effects take place through the classic or genomic pathway, in which ERα and ERβ translocate to the nucleus to bind specific responsive elements on target genes. However, faster effects need signaling through PKA, PI3K and MAPK (Honda et al., 2000; Yamakawa and Arita, 2004). Accordingly, the ER pathway can activate p38, and reciprocally p38 can act on ER. p38 is stimulated by β-estradiol in human dental papilla cells, in which both proliferation and some odonto-osteogenic markers are enhanced, depending on JNK and p38 (Li et al., 2014b). The activation of p38 by this molecule takes place through ERα, but not ERβ (Geraldes et al., 2003; Mori-Abe et al., 2003). In contrast, in human dental pulp cells, the upregulation of OPG by estradiol is reported, and SB203580 blocks this effect. Strikingly, these actions do not seem to take place through the classic ERα and ERβ receptors, as their chemical agonists do not trigger downstream effects on OPG (Manokawinchoke et al., 2016). In MC3T3, saponin diosgenin acts on ER and, as a result, stimulates the production of VEGF via the interplay of p38, PI3K, and HIF (Yen et al., 2005). In MG-63 osteoblastic cells, β-estradiol and mechanical stress can phosphorylate ERK and p38 independently, with different kinetics. By combining both treatments, the researchers reported the synergistic expression of *Ptgs2* (encoding COX2) and *Fos* by elevating *Integrin-b1* levels (Yeh et al., 2010).

#### PTH

The first link of PTH with p38 in MC3T3 implicated the necessary activation of the cAMP-PKA pathway (Rey et al., 2007). The chronic or intermittent treatment of PTH on the beta-arrestin-2 (*Arrb2*) knockout show that the main signaling pathways implicated are p38 and NFκB (Bianchi and Ferrari, 2009). On the *Mkp1* knockout, the interplay between this phosphatase, p38 and PTH was investigated. The continuous treatment of PTH leads to effects on p38 signaling by acting on the phosphatase MKP-1, negatively regulating ERK and p38 (Mahalingam et al., 2013). PTH downregulates the cell cycle and apoptosis regulatory protein 1 (CARP1) via p38 in osteoblastic cells (Sharma et al., 2013). p38 was also needed for COX2 stimulation by PTH in primary osteoblasts (Park et al., 2007).

The most relevant study on intermittent treatment with PTH and the role of p38 has been published recently. As shown with the conditional knockout *Ocn-Cre*; *p38a*^*f*/*f*^, PTH could not exert its anabolic effects *in vivo*, due to a lack of p38 in mature osteoblasts. Although the authors reported that, in these animals, PTH-induced expression of *Rankl* is supressed, still the net effect of p38 deletion is reduced ossification, due to the inability of PTH to induce osteogenic genes. In addition, the authors establish the possibility that PTH activates p38 through cAMP/PKA signaling (Thouverey and Caverzasio, 2015b). Mechanistically, it has been proposed that PTH increases both SMAD and p38 BMP signaling in MSCs (Yu et al., 2012).

#### FGF, IGF-1, and VEGF

The first hints of p38 activation by FGF2 in MC3T3 were reported in 1997, on behalf of MMP1 expression, even before any commercial pp38 antibody was available (Newberry et al., 1997). Subsequently, it was proven that FGF2 induces p38 phosphorylation in MC3T3 (Kozawa et al., 1999), and in immortalized human neonatal calvarial cells (Debiais et al., 2001). In another report, it was suggested that strontium ranelate plays a similar role in combination with FGF2. Moreover, MAPK induction is blocked by an FGF inhibitor (Caverzasio and Thouverey, 2011). It should be noted that strontium ranelate alone stimulates p38 and ERK activation in C3H10T2, inducing proliferation (Caverzasio, 2008). In human MSC, IGF-1 activates p38, JNK, and ERK. These kinases are essential to the upregulation of Osterix upon IGF-1 treatment, as this is blocked by chemical inhibitors (Celil and Campbell, 2005). Although VEGF cannot activate p38 alone in human adipose mesenchymal stem cells, it can cooperate with BMP6 signaling to direct differentiation toward osteoblastic commitment, and to avoid adipogenesis. This positive collaboration is mediated via simultaneous activation of p38 and AKT inhibition (Li et al., 2015a).

#### AMPK/adiponectin

Adiponectin can activate p38 and JNK by binding to its own receptor, as AdipoR1 siRNAs block p38 phosphorylation in primary trabecular osteoblasts. Interestingly, the p38 and JNK cascades are independently and respectively associated with osteoblast activity and proliferation (Luo et al., 2005). Indeed, in C3H10/T2 cells, adiponectin induces osteogenic genes such as *Spp1, Bglap*, and *Ptgs2* via p38 through AdipoR1, but not AdipoR2. Also, adiponectin favors RUNX2 and CREB recruitment to *Bglap* and *Spp1* promoters. (Lee et al., 2009). Adiponectin is also seen as a p38 stimulus to favor *RANKL* transcription and at the same time inhibit *OPG*, thus leading to osteoclast activation (Luo et al., 2006). Intriguingly, regarding osteoblastogenesis, the relationship between adiponectin and p38 stimulation may generate different outputs depending on the cell type. In calcifying vascular smooth muscle cells, adiponectin p38-activation is related to impairment of osteoblastic differentiation (Luo et al., 2009).

### Inflammatory cytokines

#### Interleukins

Interleukin-1β (IL-1β) activates p38 in MG-63 and MC3T3 cells, which helps to induce IL-6 and stabilize its mRNA (Kumar et al., 2001; Patil et al., 2004). In addition, IL-1β activates both ERK and p38, but not JNK, in MG-63 cells (Lambert et al., 2007). It also accelerates osteoblastic differentiation, presumably through p38 activation, as the inhibitor SB2035820 supresses primary rat osteoblasts' mineralization (Lin et al., 2011). Conversely, one report analyzed the negative impact of IL-1β on BMP-2 osteogenesis. These effects are, at least in part, due to p38 activation by this interleukin (Huang et al., 2014). Moreover, p38 and ERK are linked to IL-1β-induced *Opg* expression, therefore presumably preventing resorption (Lambert et al., 2007).

#### Prostaglandins

In calvarial osteoblasts, PGE_2_ activates p38 via EP2 and cAMP, while ERK is triggered via EP4/PKC, and JNK can be activated by both EP2 and EP4. Inactivation of each pathway results in decreased osteogenic markers (Minamizaki et al., 2009). In MC3T3, PGE_1_ signaling through p38 and ERK increases VEGF synthesis and ALP activity. Moreover, the same authors suggested that in this cascade, p38 would be downstream of cAMP and PKA (Tokuda et al., 2001; Kakita et al., 2004). In the cell line MG-63, 15d-PGJ_2_ (15-deoxy-delta-12,14-prostaglandin J2) independently activates p38 in a very rapid manner. The use of inhibitors PD169316 and PD098580 blocks 15d-PGJ_2_-induced *PTGS2* transcription (Kitz et al., 2011). The same prostaglandin prevents cell death of this osteosarcoma line. The mechanism of action includes signaling via the p38/AKT/Egr-1 axis (Koyani et al., 2016).

#### TNFα

TNFα is recognized as an inductor of p38 and is implicated in IL6 synthesis in MSCs (Zhou et al., 2006) and in MC3T3 (Dai et al., 2006). Huang et al showed that TNFα inhibited BMP2 osteogenesis via p38. Chemical inhibition of p38 restores the expression levels of RUNX2 and other osteogenic markers triggered by BMP-2, despite the presence of TNFα (Huang et al., 2014). TNFα-mediated p38 activation has also been linked to *Rankl* early expression, but not during the late phase (Dai et al., 2006).

### ECM and adhesion

#### Rho-kinase

Endothelin-1 and prostaglandin-F_2α_ (PGF_2α_) can activate IL-6 by acting both on Rho-kinase and p38 on MC3T3. The effects disappear after treatment with the Rho inhibitors fasudil or Y27632, or the p38 inhibitors SB2035820 and BIRB0796 (Minamitani et al., 2008). Thrombin can also directly activate ERK and JNK, and indirectly activate p38 through Rho-kinase. The actions on ERK and p38 lead to an increase of IL-6 that can be stopped by fasudil and Y27632 (Kato et al., 2011). Fluid flow forces can also activate PI3K, ERK, and p38 via RhoA-ROCK in osteoblasts (Hamamura et al., 2012). Dr. Glimcher's group found that FGD1 signals through CDC42 to the MAP3K MLK3, which in turn activates p38 and ERK, provoking RUNX2 phosphorylation. Mutations in *FGD1* are associated with faciogenital dysplasia (FGDY), which is characterized by skeletal defects that are comparable to the phenotype of *Mlk3*^−/−^ mice (Zou et al., 2011).

#### Extracellular matrix proteins

The extracellular matrix holds a set of molecules that can directly activate the MAPK or are needed for MAPK function. For instance, calcium, one of the main constituents of bone ECM, elevates MC3T3 proliferation via ERK and p38 activation, as its chemical blockage interrupts the proliferative effect (Yamaguchi et al., 2000). CCNs are ECM proteins that can act as growth factors and are critical for osteoblast function. CCN-1 to -6 have a positive impact on *Runx2, Sp7, Col1a1, Alp*, and *Bglap* expression, partly due to activation of ERK and p38 (Kawaki et al., 2011). The expression of BMP4 induced by CCN3 depends on p38 and JNK function, as demonstrated with a chemical antagonist and dominant negative MAPK forms (Tan et al., 2012). Dentin matrix protein1 (DMP1) has been proven to serve as an inductor of differentiation for odontoblasts and osteoblasts. It was demonstrated that DMP-1 increases intracellular calcium levels in several osteoblastic cells, and stimulates phosphorylation and nuclear translocation of p38, which leads to activation of downstream targets such as MK2 and HSP27, and nuclear translocation of RUNX2 (Eapen et al., 2010). DDR2 (Discoidin Domain Receptor-2) is a tyrosine-kinase receptor with collagen affinity. *Ddr2* is transcriptionally activated by ATF4 and C/EBPβ, where the former is a known target of p38. At the same time, DDR2 needs p38 activity to trigger Runx2 and osteocalcin expression (Lin et al., 2010). In addition, Plexins (Plxn) are semaphorin receptors that were originally involved in cell adhesion and migration. PlxnA2 is expressed in bone, and its siRNA inhibition reduces phosphorylated levels of SMAD1, AKT, and p38, as well as Runx2 expression and mineralization. Moreover, it seems that PlxnA2 activity may be associated with binding to BMPRs (Oh et al., 2012).

### Stresses and physical inputs

#### Chemical stressors

MAPKs have been traditionally described as stress kinases that can be triggered by multiple stressors. The use of certain chemical compounds was recurrent in many early *in vitro* experiments. For example, arsenate induces p38, and this leads to an increment in peroxiredoxin I protein levels in MC3T3, but does not affect its mRNA levels (Li et al., 2002). Another chemical stressor, cadmium, requires p38 function to induce PGE_2_ in primary osteoblasts (Miyahara et al., 2004). In addition, temperature stress can also trigger p38 in MSC, which has a positive effect on their osteogenic commitment. Indeed, chemical blockage of this MAPK prevents the stimulation of proteins such as RUNX2, OPN, BSP, and collagen I (Nie et al., 2015).

#### Hypoxia

In MSCs, hypoxia clearly induces osteoblastic differentiation through ERK and p38 activation, particularly when they are seeded in bone-derived scaffolds (Zhou et al., 2013). Similar effects are seen in periodontal ligament cells when co-cultured with endothelial cells (Wu et al., 2013). Hypoxia has also been studied in osteoarthritic osteoblasts, where it upregulates leptin, which also involves p38 signaling (Bouvard et al., 2014).

#### Mechanical inputs

Mechanical loading constantly shapes the skeleton, and some physical stimuli have been shown to activate p38 in several *in vitro* and *in vivo* experimental settings. Static stretching is an *in vitro* model that tries to reproduce the effects of bone distraction on osteoblasts. In this model, p38 and ERK are phosphorylated, which stimulates VEGF release in human MSCs. When these MAPKs are inhibited, VEGF is no longer activated (Kim et al., 2010). Using the same model, other authors showed effects of p38 on BMP-2 and BMP4 production, which in turn activate *Col1a1, Runx2, Spp1, Alp*, and *Bglap* in MC3T3 (Wang et al., 2012; Zhang et al., 2013). Interestingly, siRNAs against TAK1 dramatically soften this stretching activation in MC3T3, as well as IL-6 expression, which is also decreased by blocking p38 (Fukuno et al., 2011). ROS17/2.8 cells subjected to stretching or to microgravity show that inhibition of p38 by SB203580 extended the time expression of *Egr1* (Granet et al., 2001). Physiologically, stretching forces also play a role in the periodontal space. Indeed, several reports show that cyclic tension activates MAPK signaling and increases osteogenesis in human periodontal ligament cell (Li et al., 2014a; Suzuki et al., 2014). In this model, p38 and ERK activation, but not JNK, are related to *PTGS2* and *BMP2* transcription (Suzuki et al., 2014).

Furthermore, fluid flow shear stress can also activate p38 and ERK in bone marrow stromal cells (Kreke et al., 2008) and in MG63 (Lee et al., 2008a). The activation of these MAPKs is associated with the interplay between integrins a_*v*_β_3_ and β1 with the adaptor protein Shc. This leads to *Fos, Ptgs2*, and *Spp1* expression (Lee et al., 2008a). Integrins can also activate p38 by contacting other molecules. In human osteosarcoma cells, angiopoietin-like protein 2 (ANGPTL2) appears to interact with integrin α_5_β_1_ to promote p38 activation and *MMP9* expression, which favors invasiveness (Odagiri et al., 2014). Fluid flow cell stress in MC3T3 activates ERK and p38 via Rho kinase, leading to expression of *Ptgs2, Spp1*, and *Per2* (Hamamura et al., 2012).

#### Ultrasound, electric and magnetic fields and lasers

Other osteogenic stimuli that are not related to loading mechanical forces but electromagnetic waves also have osteogenic potential via p38 (Xiao et al., 2009). Ultrasounds can stimulate MAPKs in primary osteoblasts cultures and favor the expression of metalloproteinase 13 (MMP13), which increases the binding of Fos and Jun to AP-1 elements at promoter level. These effects are supressed by treatment with p38 inhibitors SB2035820 or JNK inhibitor SP600125, but not when ERK is inhibited (Chiu et al., 2008). Previously, it was shown that p38 inhibitor SB203580 prevents ultrasound-induced COX2 and osteocalcin expression in murine cells ST2 (Naruse et al., 2003). Electromagnetic stimulation increases p38, JNK, and ERK phosphorylation levels in MC3T3. Inhibition of these MAPK is related to a reduction in electromagnetically induced osteopontin, PDGF and VEGF levels (Yumoto et al., 2015). Similarly, low frequency electromagnetic fields favor collagen accumulation, probably via p38, as the use of SB203580 diminishes this effect (Soda et al., 2008). In addition, high frequency fields raise p38 mRNA levels in C3H10T2 (Teven et al., 2012). Biphasic electric current has also been applied in an *in vitro* osteogenic model with human stromal cells. This stimulus activates both ERK and p38 MAPK pathways, leading to increased proliferation and induction of VEGF and HIF-α, which can be supressed by chemical inhibition (Kim et al., 2009).

### Pharmacological compounds

Bisphosphonates were found to induce ERK and p38 pathways, halt proliferation, and favor apoptosis in sarcoma cell lines (Kubo et al., 2008). Statins, therapeutic drugs to fight hypercholesterolemia, can have a positive effect on bone formation. Simvastatin induces HSP27 specifically via p38 signaling in MC3T3, and SB203580 blocks this effect (Wang et al., 2003). Generally, statins and bisphosphonates can have a dual action on bone cells: they can inhibit the ERK pathway, and accentuate p38 signaling. ERK and p38 seem to be reciprocally inhibited. The activation of p38 favors OPG overexpression, while the inhibition of ERK downregulates CD-1, RANKL, and MCSF. Thus, the net result of bisphosphonate and statin treatment would be inhibition of osteoblast-induced osteoclast maturation (Tsubaki et al., 2012). Thiazolidinediones (TZD), a drug for diabetes type 2 treatment, can also stimulate ERK and p38 via GPR40 and Ras activation. Apparently, this pathway, which is independent of PPARγ activity, leads to TZD-induced osteocyte apoptosis (Mieczkowska et al., 2012).

In traditional Asian medicine, a myriad of herb and plant compounds have been used historically to treat bone diseases such as osteoporosis or fractures. Many of these compounds contain remarkable doses of pro-estrogenic chemicals, antioxidants or anti-inflammatory agents, and have been linked to p38 function. Phytoestrogens (isoflavonoids) group non-steroidal compounds of plant origin that resemble human estrogens. Some of them have been studied as bone anabolic therapies, as they increment the levels of several osteogenic markers. They include genistein (which involve a p38-RUNX2), 8-prenylkaempferol, sesamin and caffeic acid 3,4-dihydroxy-phenethyl ester (CADPE) (Chiou et al., 2011; Wanachewin et al., 2012; Liao et al., 2014; Schilling et al., 2014). Another important group of natural substances is the flavonoid family. Flavonoids are present in fruit, vegetables and seeds, and have long been known for their beneficial effects. Naringin, ugonin K, neobavaisoflavone, puerarin, quercetin, icariin and apigenin have shown anabolic abilities in some osteogenic models both *in vivo* and *in vitro* (Ming et al., 2013; Welch and Hardcastle, 2014; Li et al., 2015b; Zhang et al., 2015; An et al., 2016). The saponins family also has proven *in vitro* osteogenic actions that affect both osteoblast proliferation and differentiation (Jeong et al., 2010; Niu et al., 2011; Zhou et al., 2015) and osteoclast activation (Zhou et al., 2015). Other substances, such as coumarinic compounds, have only been associated with osteoblast commitment (Tang et al., 2008). Lactone derivatives affect both p38 and ERK pathways and are linked to osteoblast function (Lee and Choi, 2011) and osteoclast impairment (Zhai et al., 2014). Isoquinolines, quinones and lignans can increase ALP activity and *Bmp2* expression, as well as upstream effectors like RUNX2, in pre-osteoblasts and mesenchymal cells and *in vivo* models (Lee et al., 2008b; Kim et al., 2014; Moon et al., 2015; Yang et al., 2015a). In addition, phenolic compounds are associated with positive effects on osteogenesis. A diet enriched in blueberries seems to ameliorate bone marker levels and diminish bone resorption in rats. These changes are related to p38 activation and high levels of beta-catenin (Chen et al., 2010). Xanthonoids in gambogic acid are linked to decreased osteoclastogenesis and bone loss (Ma et al., 2015).

## Concluding remarks

p38 MAPK plays a pivotal role in different steps of osteoblast differentiation. *In vivo*, p38 deletion hampers osteoblast terminal differentiation and the appearance of osteocytes, which directly affects bone composition and maintenance. Taking into account the growing number of triggers of p38 activity and the key action of MAPK in bone development and homeostasis, we should consider p38 as a central hub for signaling convergence toward osteoblastogenesis. As seen in multiple *in vitro* cell-based models, p38 integrates inputs from different stimuli. The latter range from mechanical loading to signaling molecules like cytokines, of which BMP/TGFβ and Wnt pathways have been the most widely studied. The *in vivo* evidence reinforces this hypothesis. As seen in specific knockouts, deleting some key effectors of the p38-MAPK pathway affects osteoblast development at different moments during differentiation. Therefore, disturbing p38 affects a necessary integrator of various signaling inputs.

Matrix deposition and shaping is a local, fine-tuned event affected by multiple factors. We know that MAPKs such as p38, ERK, and JNK are rapidly activated, and are thus convenient for an anabolic cell like the osteoblast to respond to different local spurs. Bearing all this in mind, the p38 cascade could be a good target for anabolic bone therapies. However, the large number of activators and the different levels of self- and cross-regulation make it difficult to specifically target it therapeutically. On another front, assessing p38 activity could inform clinicians about the ability of bone to respond to anabolic therapies.

## Author contributions

ER, BG, and FV conceived, analyzed and discussed the manuscript. ER and FV wrote the manuscript. ER and BG draw the figures.

## Funding

This work was supported by grants from the MEC (BFU2014-56313P) and Fundació La Marató de TV3.

### Conflict of interest statement

The authors declare that the research was conducted in the absence of any commercial or financial relationships that could be construed as a potential conflict of interest.
